# Effects of Hydrogen Peroxide on Different Toxigenic and Atoxigenic Isolates of *Aspergillus flavus*

**DOI:** 10.3390/toxins7082985

**Published:** 2015-08-05

**Authors:** Jake C. Fountain, Brian T. Scully, Zhi-Yuan Chen, Scott E. Gold, Anthony E. Glenn, Hamed K. Abbas, R. Dewey Lee, Robert C. Kemerait, Baozhu Guo

**Affiliations:** 1 Department of Plant Pathology, University of Georgia, Tifton, GA 31793, USA; E-Mails: jfount1@uga.edu (J.C.F.); kemerait@uga.edu (R.C.K.); 2 USDA-ARS, Crop Protection and Management Research Unit, Tifton, GA 31793, USA; 3 USDA-ARS, U.S. Horticultural Research Laboratory, Fort Pierce, FL 34945, USA; E-Mail: brian.scully@ars.usda.gov; 4 Department of Plant Pathology and Crop Physiology, Louisiana State University, Baton Rouge, LA 70803, USA; E-Mail: zchen@agcenter.lsu.edu; 5 USDA-ARS, Toxicology and Mycotoxin Research Unit, Athens, GA 30605, USA; E-Mails: scott.gold@ars.usda.gov (S.E.G.); anthony.glenn@ars.usda.gov (A.E.G.); 6 USDA-ARS, Biological Control of Pests Research Unit, Stoneville, MS 38776, USA; E-Mail: hamed.abbas@ars.usda.gov; 7 Department of Crop and Soil Sciences, University of Georgia, Tifton, GA 31793, USA; E-Mail: deweylee@uga.edu

**Keywords:** aflatoxin, drought, oxidative stress, reactive oxygen species, biological controls, peptone

## Abstract

Drought stress in the field has been shown to exacerbate aflatoxin contamination of maize and peanut. Drought and heat stress also produce reactive oxygen species (ROS) in plant tissues. Given the potential correlation between ROS and exacerbated aflatoxin production under drought and heat stress, the objectives of this study were to examine the effects of hydrogen peroxide (H_2_O_2_)-induced oxidative stress on the growth of different toxigenic (+) and atoxigenic (−) isolates of *Aspergillus flavus* and to test whether aflatoxin production affects the H_2_O_2_ concentrations that the isolates could survive. Ten isolates were tested: NRRL3357 (+), A9 (+), AF13 (+), Tox4 (+), A1 (−), K49 (−), K54A (−), AF36 (−), and Aflaguard (−); and one *A. parasiticus* isolate, NRRL2999 (+). These isolates were cultured under a H_2_O_2_ gradient ranging from 0 to 50 mM in two different media, aflatoxin-conducive yeast extract-sucrose (YES) and non-conducive yeast extract-peptone (YEP). Fungal growth was inhibited at a high H_2_O_2_ concentration, but specific isolates grew well at different H_2_O_2_ concentrations. Generally the toxigenic isolates tolerated higher concentrations than did atoxigenic isolates. Increasing H_2_O_2_ concentrations in the media resulted in elevated aflatoxin production in toxigenic isolates. In YEP media, the higher concentration of peptone (15%) partially inactivated the H_2_O_2_ in the media. In the 1% peptone media, YEP did not affect the H_2_O_2_ concentrations that the isolates could survive in comparison with YES media, without aflatoxin production. It is interesting to note that the commercial biocontrol isolates, AF36 (−), and Aflaguard (−), survived at higher levels of stress than other atoxigenic isolates, suggesting that this testing method could potentially be of use in the selection of biocontrol isolates. Further studies will be needed to investigate the mechanisms behind the variability among isolates with regard to their degree of oxidative stress tolerance and the role of aflatoxin production.

## 1. Introduction

The contamination of agricultural products with aflatoxins produced by *Aspergillus flavus* poses a serious threat to human health and food security, particularly in developing countries [1,2]. Research into aflatoxin contamination prevention began in the 1960s following the outbreak of what was termed turkey X disease which resulted in the deaths of over 100,000 turkey poults due to aflatoxin contaminated feed [3]. These efforts were intensified in the area of pre-harvest host resistance following outbreaks in US maize in the 1970s, and their importance has been further underscored by the 2004 Kenya outbreak which resulted in 125 human deaths due to direct aflatoxicosis from consumption of contaminated maize [4,5].

The contamination of oilseed crops, such as maize and peanut, with aflatoxin has been shown to be exacerbated by the presence of drought stress and related abiotic stresses [6,7]. Host drought tolerance and reduced aflatoxin accumulation are also correlated [6,7]. Given this observation, it is important to better understand the mechanisms at play in this relationship and how they may relate to both environmental stress tolerance and to the regulation of aflatoxin production in the pathogen.

Aflatoxin production in the *Aspergilli* is a complex process under a high degree of regulation through multiple mechanisms [8,9]. Numerous studies have shown that aflatoxin production can be exacerbated by reactive oxygen species (ROS) and their reactive products including oxylipins [10,11]. For example, Jayashree and Subramanyam [12] demonstrated that peroxidized lipids can stimulate the production of aflatoxin in *A. parasiticus* and accumulate to high levels during trophophase growth which coincides with aflatoxin production initiation in toxigenic isolates. Additional studies have also demonstrated that toxigenic isolates exhibit elevated oxygen consumption, greater mycelial ROS accumulation, and greater peroxisome number than atoxigenic isolates, indicating a reasonable link between ROS and aflatoxin accumulation [12,13,14,15,16]. Recent molecular studies have also revealed that aflatoxin production is regulated by stress-related transcription factor pathways mediated by AtfB and AP-1, as well as the Velvet A (VeA) signaling pathway in response to *in vitro* applied ROS [14,15,16]. The growth and development of *A. flavus* is also regulated by ROS. For example, Grintzalis *et al.* [17] demonstrated that ROS can regulate the production of aflatoxin and sclerotial differentiation in a concentration-dependent manner.

This proposed link between ROS and aflatoxin production has several implications with regard to the pathogen’s biology and host defense against aflatoxin contamination. Given the correlation observed between elevated aflatoxin production and ROS accumulation, it has been proposed that aflatoxin production or associated mechanisms may provide some advantages in the drought and heat stress environments with high ROS to compete with other microorganisms [16,18,19,20]. This opens the possibility that ROS may also function in the host-pathogen interaction between *Aspergillus* spp. and their host plants as a form of “cross-kingdom communication” [16,19]. This seems plausible given the observed correlation between drought stress, which results in the accumulation of ROS in host plant tissues, and the exacerbation of aflatoxin contamination in oilseed crops such as maize and peanut [6,7,21,22].

Given this correlation among drought and heat stresses, ROS and aflatoxin production, it is possible that the ability to produce aflatoxin may influence the growth of *Aspergillus* spp. isolates when exposed to drought stress-derived ROS such as hydrogen peroxide (H_2_O_2_) Therefore, the objectives of this study were twofold: to examine the effects of H_2_O_2_-induced oxidative stress on the growth of different isolates of toxigenic (+) and atoxigenic (−) isolates of *Aspergillus flavus*, and to test whether aflatoxin production affects the concentrations of H_2_O_2_ in which the isolates could grow and survive. To test this, the differences in oxidative stress tolerance between toxigenic (+) and atoxigenic (−) isolates were examined using hydrogen peroxide (H_2_O_2_) as oxidative stress encountered during drought stress in both aflatoxin conducive and non-conducive media. Overall, for the isolates tested, toxigenic isolates generally tolerated higher levels of oxidative stress than did atoxigenic isolates. The reduction in aflatoxin production did not affect the survival levels of ROS induced by supplemental H_2_O_2_ in aflatoxin non-conducive 1% peptone YEP media. In addition, selected commercially available atoxigenic biological control isolates were able to tolerate greater levels of stress than non-selected isolates indicating the potential utility of this experimental method in screening local atoxigenic isolates for use as biological control agents in developing countries [23].

## 2. Results and Discussion

### 2.1. Oxidative Stress Tolerance in Toxin-Conducive Media

In order to determine the effects of oxidative stress encountered during drought on toxigenic and atoxigenic isolates, we simulated this stress *in vitro* using H_2_O_2_ supplemented aflatoxin production conducive media. The applied concentrations of H_2_O_2_ were based on those utilized in previous studies [14] and based on the range of hydrogen peroxide levels previously observed to be cytotoxic to plant cells [24,25].

In the aflatoxin production-conducive 15% sucrose YES media at the higher concentrations of supplemental H_2_O_2_, fungal growth was inhibited, but the different isolates grew well at different concentrations of H_2_O_2_. The toxigenic isolates including *A. flavus* Tox4, A9, AF13, and NRRL3357; and *A. parasiticus* NRRL2999 were able to grow well at levels of oxidative stress up to 40, 40, 35, 25, and 25 mM H_2_O_2_, respectively (Table 1). The atoxigenic isolates including *A. flavus* K49, AF36, Aflaguard, A1, and K54A, however, were only able to survive oxidative stress up to 30, 25, 25, 20, and 15 mM H_2_O_2_, respectively (Table 1). This indicates that there is a great deal of variability among the isolates with regard to their degree of oxidative stress tolerance with or without aflatoxin production in the natural environment.

**Table 1:** Average dry weights (g) of *A. flavus* mycelia in H_2_O_2_ amended yeast extract-sucrose (YES) (15% sucrose) media.

Isolate	Toxin	Hydrogen Peroxide Concentrations (mM)										
		0	5	10	15	20	25	30	35	40	45	50
Tox4	+	1.08	1.10	1.13	1.09	1.07	1.05	1.05	1.06	1.09	0	0
A9	+	1.14	1.12	1.12	1.10	1.14	1.15	0.80	0.99	0.49	0	0
AF13	+	1.15	1.13	1.12	1.14	1.08	1.09	1.11	1.19	0	nt	nt
NRRL3357	+	1.17	1.14	1.12	1.11	0.64	0.34	0	nt	nt	nt	nt
NRRL2999	+	1.12	1.11	1.21	1.06	1.03	1.19	0	nt	nt	nt	nt
K49	−	1.12	1.11	1.09	1.11	0.88	0.90	0.47	nt	nt	nt	nt
AF36	−	1.19	1.17	1.14	1.11	0.95	1.07	0	nt	nt	nt	nt
Aflaguard	−	1.16	1.12	1.15	1.19	0.79	0.38	0	nt	nt	nt	nt
A1	−	1.15	1.15	1.13	1.21	0.82	0	0	nt	nt	nt	nt
K54A	−	1.12	1.16	1.11	0.81	0	0	0	nt	nt	nt	nt

Note: nt: not tested; +: toxigenic; −: atoxigenic.

**Figure 1:**
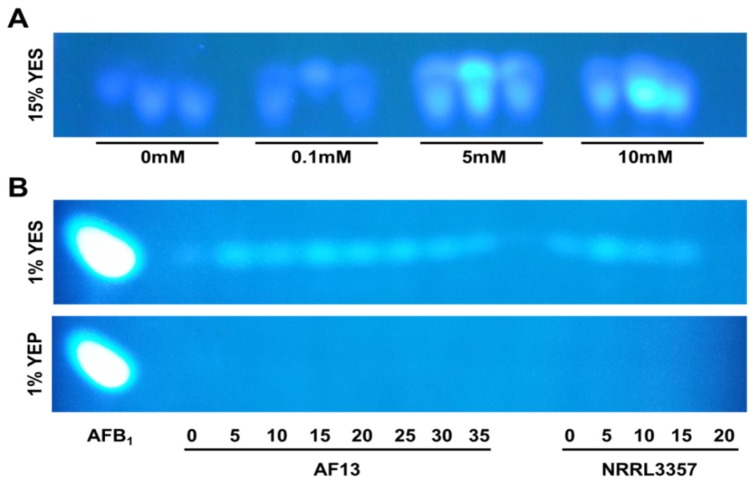
Aflatoxin production of select *A. flavus* isolates under H_2_O_2_-induced oxidative stress in toxin-conducive yeast extract-sucrose (YES) and non-conducive yeast extract-peptone (YEP) media visualized using thin layer chromatography (TLC). (**A**) Isolate NRRL3357 was cultured in YES media containing 15% sucrose and supplemented with H_2_O_2_ ranging from 0 (control check) to 10 mM. Increasing visible fluorescence over the concentration gradient indicates elevated levels of aflatoxin B_1+2_. (**B**) Isolates AF13 and NRRL3357 were cultured on YES and YEP media containing reduced carbon source concentrations (1%). Elevated aflatoxin production was observed in response to oxidative stress in 1% YES media (compared to “0” control) while no aflatoxin production was observed in 1% YEP medium. An aflatoxin B_1_ standard was included as a reference.

However, the toxigenic isolate NRRL3357 survived only to 25 mM H_2_O_2_, a level comparable to most of the atoxigenic isolates examined in the study. Previous studies have shown that toxigenic isolates accumulate elevated levels of aflatoxin in response to increasing oxidative stress *in vitro* [12,13]. This was also observed here in the toxigenic isolates with increasing levels of H_2_O_2_-induced stress resulting in elevated levels of aflatoxin production in the toxigenic isolates grown in toxin-conducive 15% YES media (Figure 1A). These results are similar to those observed by Roze *et al.* [26] where isolates produced greater levels of aflatoxin with increased levels of oxidative stress. Nonetheless, the production of aflatoxin as observed in NRRL3357 was lower in comparison to other examined isolates. Therefore, it is likely that aflatoxin production alone may not directly contribute to the stress tolerance in toxigenic isolates in general as observed here but other factors which are in need of further investigation.

Additional studies have also shown that toxigenic isolates exhibit enhanced antioxidant enzyme activities during the initiation of aflatoxin production, which may enhance the oxidative stress tolerance of the isolates [12,13]. Narasaiah *et al.* [20] suggested that the production of aflatoxin biosynthetic pathway intermediates may also function to remediate oxidative stress through the consumption and sequestration of ROS during their biosynthesis. However, the specific roles of these compounds in oxidative stress responses of *A. flavus* isolates have yet to be demonstrated. This apparent co-expression of antioxidant enzymes and aflatoxin production in response to H_2_O_2_-induced oxidative stress can have two possible interpretations: (1) aflatoxin production can function in further enhancing oxidative stress tolerance beyond that afforded by the antioxidant enzymes alone; or (2) aflatoxin and/or pathway intermediates may cause additional oxidative stress for as yet unknown reasons, and antioxidant enzymes are elevated in activity to counter this stress [18]. Continuing studies in the laboratory will examine the effects of these intermediate compounds and aflatoxin in enhancing or diminishing oxidative stress tolerance in *Aspergillus* spp.

Another trend was observed in the atoxigenic isolates. It was found that the atoxigenic isolates exhibiting the highest levels of oxidative stress tolerance were the K49, AF36, and Aflaguard biological control isolates (Table 1). These isolates have been selected due to their adaptation to local environments in the U.S., and their performance as effective biological controls for remediating aflatoxin contamination in maize and peanut [27,28,29].

Since environmental stress adaptation and competitive capabilities against toxigenic isolates likely are key indicators of biocontrol isolate performance, the selection of local adapted atoxigenic isolates for use as biological controls is preferred to introducing non-adapted isolates [23]. This concept has been demonstrated in sub-Saharan Africa with the selection of local adapted atoxigenic isolates for use as biological controls, and through the utilization of blends of isolates to enhance the diversity of the biological controls for broader efficacy in remediating aflatoxin contamination [23,30,31].

Given the need, therefore, to rapidly and efficiently screen atoxigenic isolates for utility as biological control agents, an effective, low cost, and technically simple method is needed. Several available screening methods are currently used based on competitive inhibition of aflatoxin production in which potential biological control isolates are co-inoculated with toxigenic isolates onto maize kernels [31], or into coconut liquid media [32]. These methods examine the effectiveness of potential biological control isolates in inhibiting aflatoxin production by toxigenic isolates, but do not evaluate their environmental stress tolerance, which is necessary if the isolate will be deployed in multiple, non-native environments. Since H_2_O_2_-induced oxidative stress occurs in organisms in response to abiotic stresses such as drought and heat [33], it is possible that the method used in this study could be utilized to screen potential biological control isolates for environmental stress tolerance *in vitro* prior to deployment in field trials. This seems plausible given that atoxigenic isolates that have been selected for their performance as biological controls and permanence in the field (K49, AF36, and Aflaguard) possess elevated oxidative stress tolerance in comparison to non-selected atoxigenic isolates (A1 and K54A). Continuing studies in the laboratory will also examine the potential of this H_2_O_2_ gradient method in predicting atoxigenic isolate performance.

### 2.2. Effects of Carbon Source on Aflatoxin Production and Isolate Oxidative Stress Tolerance

In order to examine the role of aflatoxin production in oxidative stress responses, the isolates were cultured in a toxin non-conducive media, 15% peptone YEP, under the same conditions used previously with the toxin-conducive 15% sucrose YES media. If aflatoxin production functions at least in part in oxidative stress responses, it would be expected that the oxidative stress tolerance of the toxigenic isolates would be affected in non-conducive YEP media supplemented with H_2_O_2_ while the atoxigenic isolates would not be significantly affected. However, it was found that culturing the isolates in 15% peptone YEP media increased the levels of H_2_O_2_ that both the toxigenic and atoxigenic isolates could tolerate in comparison to their observed growth in 15% sucrose YES (Table 1 and Table 2). Specifically, the toxigenic isolates Tox4, A9, AF13, NRRL3357, and NRRL2999 were able to survive up to 50, 50, 50, 40, and 30 mM H_2_O_2_, respectively, and the atoxigenic isolates Aflaguard, A1, K49, AF36, and K54A were able to survive up to 45, 40, 35, 35, and 30 mM H_2_O_2_, respectively (Table 2).

Unexpectedly, modifying the carbon source availability in the culture media enhanced the survival of toxigenic isolates of *Aspergillus* spp. However, the additional enhancement of the tolerance of the atoxigenic isolates to higher concentrations of H_2_O_2_ indicates that there is a medium-specific response that may be affecting the results. In addition, the survival of the isolates at H_2_O_2_ concentrations potentially higher than 50 mM seems unlikely given the previously described oxidative stress tolerance of plant apoplasts with cell death occurring within 24 h in cultured *Arabidopsis thaliana* cells exposed to comparable levels of H_2_O_2_ [24]. Therefore, it is possible that peptone may act as an “inactivator or chelator” of H_2_O_2_ when utilized as a medium carbon source.

**Table 2:** Average dry weights (g) of *A. flavus* mycelia in H_2_O_2_ amended yeast extract-peptone YEP (15% peptone) media.

Isolate	Toxin	Hydrogen Peroxide Concentrations (mM)										
		0	5	10	15	20	25	30	35	40	45	50
Tox4	+	1.55	nt	nt	nt	1.18	1.21	1.18	1.03	1.50	1.28	1.04
A9	+	2.21	nt	nt	nt	1.18	1.19	1.09	1.14	1.42	1.31	1.33
AF13	+	1.38	1.35	1.38	1.4	1.37	1.41	1.32	1.29	1.24	1.39	1.28
NRRL3357	+	1.39	1.35	1.52	1.33	1.30	1.04	0.80	0.78	0.83	0	nt
NRRL2999	+	1.70	nt	nt	nt	1.15	0.98	0.71	0	0	0	nt
K49	−	1.09	1.06	1.09	1.08	1.16	0.74	0.65	0.73	0	0	nt
AF36	−	1.40	nt	nt	nt	1.34	1.02	0.80	0.62	0	0	nt
Aflaguard	−	1.65	nt	nt	nt	1.14	0.98	0.75	0.58	1.36	1.13	nt
A1	−	1.53	nt	nt	nt	1.01	0.93	0.77	0.30	0.76	0	nt
K54A	−	1.05	1.07	1.04	1.06	1.05	1.00	0.74	0	0	0	nt

Note: nt: not tested; +: toxigenic; −: atoxigenic.

**Figure 2:**
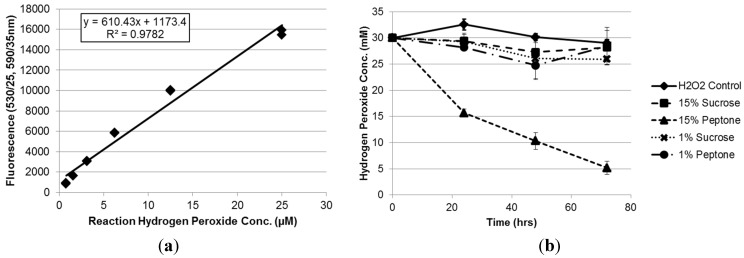
Quantification of H_2_O_2_ levels in non-inoculated culture media over time. (**a**) A standard curve was generated using stabilized H_2_O_2_ to quantify the H_2_O_2_ concentration in the media samples. (**b**) The concentration of H_2_O_2_ in non-inoculated YES and YEP culture media containing 15% or 1% carbon sources and initially supplemented to 30 mM H_2_O_2_ was monitored every 24 h for three days. The H_2_O_2_ concentration declined slowly over time in the YES media, the 1% peptone YEP media, and the water control, but plummeted sharply in YEP medium with 15% peptone (82.8% reduction in 72 h). This indicates that peptone molecules can potentially react with or inactivate H_2_O_2_.

To determine whether or not changing the culture medium carbon source introduced artifacts into the system, we measured the concentration of H_2_O_2_ in non-inoculated culture media supplemented with 30 mM H_2_O_2_ over time with carbon source concentration of 15% and a minimum concentration of 1% under the same conditions used in the previous experiments and in earlier studies by Davis *et al.* [34]. It was found that yeast extract media supplemented with sucrose (YES) at either 1% or 15% concentrations, or with peptone (YEP) at a concentration of 1% exhibited a slow rate of H_2_O_2_ degradation over time not significantly different from that observed in a water control supplemented with 30 mM H_2_O_2_ implying naturally expected H_2_O_2_ degradation in solution (Figure 2). However, when the yeast extract media was supplemented with peptone at a 15% concentration, the detected H_2_O_2_ levels in the media reduced rapidly to 52.3% and 17.2% of initial levels within 24 h and 72 h, respectively in the absence of *A. flavus* inoculum (Figure 2b).

These results indicate that elevated levels of peptone in yeast extract-based culture media can inhibit H_2_O_2_ from supplemented media thereby reducing the oxidative stress experienced by the fungi cultured in the media. This is especially true when peptone is present in higher numbers of molecules than H_2_O_2_ as seen in the present study (H_2_O_2_ to peptone peptide ratio of 2:5 in 15% YEP and 6:1 in 1% YEP assuming average peptide size of 2 kDa). This implies that the inactivating capabilities of peptone may be concentration dependent with higher levels of peptone being able to sequester more H_2_O_2_ than lower levels of peptone. If this is the case, it would be expected that if isolates were cultured at higher peptone concentrations, they would experience less H_2_O_2_-derived oxidative stress over a shorter time period resulting in the appearance of enhanced stress tolerance. Given that this system serves as the basis of suppressing aflatoxin production in order to examine the effects of oxidative stress in some molecular studies [15], it is important to consider the potential of the peptide fragments found in peptone to react with H_2_O_2_ molecules to prevent experimental bias in future studies.

### 2.3. Isolate Responses in Reduced Carbon Source Media

In order to verify that the increase in isolate survival in 15% peptone YEP was due to the sequestration of H_2_O_2_ when peptone was present in excess, we cultured select isolates in yeast extract media supplemented with either sucrose or peptone at a minimum concentration of 1%. For the 1% sucrose YES media, the selected atoxigenic isolates, AF13, NRRL3357, Aflaguard, and K54A, were able to survive H_2_O_2_ concentrations up to 35, 20, 20, and 15 mM, respectively (Table 3), which were comparable to the results observed in the 15% sucrose YES media (Table 1). For the 1% peptone YEP media, the select isolates were able to survive H_2_O_2_ concentrations up to 30, 20, 20, and 15 mM, respectively (Table 4), which was drastically reduced in comparison to the results observed in 15% peptone YEP media (Table 2). This combined with the observed reduction in H_2_O_2_ concentration in non-inoculated 15% peptone YEP media confirms that peptone sequestration of H_2_O_2_ is the likely cause of the enhanced isolate survival observed in 15% peptone YEP.

**Table 3:** Average dry weights (g) of *A. flavus* mycelia in H_2_O_2_ amended YES (1% sucrose) medium.

Isolate	Toxin	Hydrogen Peroxide Concentrations (mM)												
		0	5	10	15	20	25	30	35	40	45	50	55	60
AF13	+	0.52	0.54	0.56	0.51	0.55	0.54	0.50	0.38	0	0	0	0	0
NRRL3357	+	0.45	0.47	0.53	0.46	0.12	0	0	0	0	0	0	0	0
Aflaguard	−	0.56	0.55	0.55	0.49	0.30	0	0	0	0	0	0	0	0
K54A	−	0.30	0.33	0.40	0.16	0	0	0	0	0	0	0	0	0

Note: +: toxigenic; −: atoxigenic.

**Table 4:** Average dry weights (g) of *A. flavus* mycelia in H_2_O_2_ amended YEP (1% peptone) medium.

Isolate	Toxin	Hydrogen Peroxide Concentrations (mM)												
		0	5	10	15	20	25	30	35	40	45	50	55	60
AF13	+	0.27	0.28	0.25	0.27	0.28	0.29	0.26	0	0	0	0	0	0
NRRL3357	+	0.24	0.25	0.33	0.28	0.08	0	0	0	0	0	0	0	0
Aflaguard	−	0.27	0.31	0.28	0.29	0.22	0	0	0	0	0	0	0	0
K54A	−	0.12	0.15	0.16	0.15	0	0	0	0	0	0	0	0	0

Note: +: toxigenic; −: atoxigenic.

Comparing the responses of the isolates in the minimum carbon source media, several observations can be made. First, the level of H_2_O_2_ -induced stress that the isolates are able to tolerate is similar between the media. This is interesting given that aflatoxin production is stimulated in response to increasing oxidative stress in the toxigenic isolates in the 1% YES media as observed in the 15% YES media while no aflatoxin is produced by the isolates cultured in 1% YEP media (Figure 1B). Second, while the difference between the isolates’ response in the 1% carbon media is not pronounced, there are some slight differences particularly in the AF13 and NRRL3357 isolates. In both the 15% and 1% YES media, AF13 was able to tolerate 35 mM H_2_O_2_, but, in the 1% YEP medium, the isolate is only able to tolerate 30 mM H_2_O_2_ (Table 1, Table 3 and Table 4). In addition, the absence of observable aflatoxin production in the NRRL3357 isolate in 1% YES supplemented with 20 mM H_2_O_2_ (Figure 1B) coincides with a reduction in fungal biomass in comparison to the lower H_2_O_2_ concentrations (Table 3 and Table 4). While the reduction in aflatoxin production in 1% YEP media may result in a slight reduction in oxidative stress tolerance in the isolates, further studies will be needed to validate and determine the precise role of aflatoxin production in such responses.

**Figure 3:**
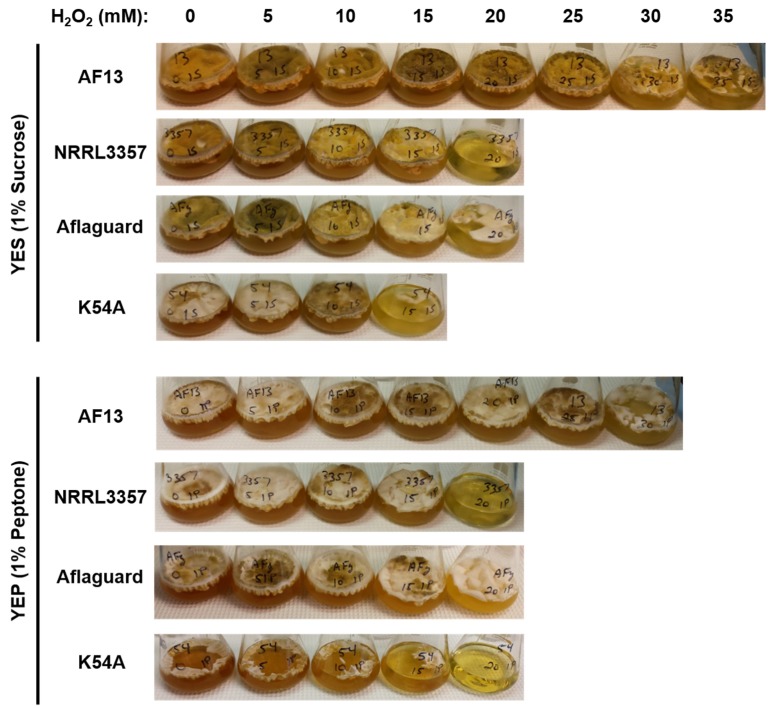
Growth behavior of selected *Aspergillus flavus* isolates under H_2_O_2_-induced oxidative stress in 1% carbon source yeast extract-sucrose (YES) and yeast extract-peptone (YEP) media. Isolates AF13, NRRL3357, Aflaguard, and K54A were cultured in 1% carbon source media and photographed for observation of their growth behavior in response to increasing H_2_O_2_ concentrations. Increased conidiation was observed from 5 to 15 mM with concentrations >15 mM resulting in reduced conidiation. Reduced growth and conidiation in YEP is likely due to reduced energy availability of carbohydrate.

The culture media also had an effect on fungal isolate development. The select isolates cultured in 1% sucrose YES medium had greater levels of conidiation than those cultured in 1% peptone YEP medium. In addition, increasing H_2_O_2_ concentrations to 5–10 mM also resulted in elevated conidiation in both media with a more pronounced effect observed in the YEP medium (Figure 3). Higher concentrations (>15 mM), however, resulted in reduced conidiation and growth (Figure 3). These results are consistent with previous observations that elevated ROS accumulation in *A. flavus* results in enhanced conidiation [35]. Overall, less conidiation was observed in YEP cultured isolates possibly indicative of delayed development in comparison to YES cultured isolates. While this slower growth is likely due to reduced energy availability resulting from the use of peptides rather than sucrose as a carbon source, further studies are needed to better understand the isolate specific responses present in either medium.

### 2.4. Summary and Future Directions

The different isolates responded to the H_2_O_2_ gradient differently in terms of biomass and survivability. The isolates survived different concentrations of H_2_O_2_-induced oxidative stress with toxigenic isolates generally tolerating higher levels of oxidative stress than atoxigenic isolates regardless of culture conditions. Aflatoxin production was also found to be enhanced by increasing H_2_O_2_ concentrations. Using peptone as the carbon source in the growth media may possibly act as an “inactivator or chelator” of H_2_O_2_. This simple assay may also be used as a screening assay for local native biocontrol isolates of atoxigenic *A. flavus.*

Given these findings, future studies examining the oxidative stress responses of *Aspergillus* spp. using this media system should take into account the potential experimental bias associated with using elevated carbon source concentrations. In addition, further studies are needed to better characterize the role of aflatoxin production in the elevated oxidative stress caused by drought and heat stress in the field, which may afford *Aspergillus* spp. an advantage in competition with other microorganisms. The initial ROS caused by drought stress in the field may further induce the production of aflatoxin by the fungus that might accelerate increases in ROS levels resulting in potential damage to the fungus itself [26]. Since it has been demonstrated that drought and related abiotic stresses result in the accumulation of ROS in the tissues of maize lines which are susceptible to aflatoxin contamination while resistant maize lines accumulate less ROS in their tissues, the role of ROS, specifically H_2_O_2_, should be investigated as potential signaling molecules in the maize-*A. flavus* interaction [18,21,22].

## 3. Experimental Section

### 3.1. Isolate Collection

The isolates of *A. flavus* and *A. parasiticus* utilized in the study were collected from various sources as follows. The *A. flavus* isolate NRRL3357 and the *A. parasiticus* isolate NRRL2999 were obtained from the Northern Regional Research Center, USDA-ARS, Peoria, IL. The *A. flavus* isolates K49 and K54A were obtained from Dr. Hamed Abbas, USDA-ARS Biological Control of Pests Research Unit (Stoneville, MS, USA). The *A. flavus* isolates A1, A9, AF13, AF36, Aflaguard, and Tox4 were obtained from Dr. Kenneth Damann, Department of Plant Pathology and Crop Physiology, Louisiana State University (Baton Rouge, LA, USA). All isolates were shipped on potato dextrose agar (PDA) and were re-cultured on V8 agar (20% V8, 1% CaCO_3_, 3% agar).

### 3.2. Culture Conditions and Biomass Measurement

The isolates were cultured on V8 agar plates at 32 °C from 5 to 7 days prior to use in the experiment. Conidia were then harvested from the plates in sterile water with 0.1% (*v*/*v*) Tween 20 and stored at 4 °C for further use. Four media were utilized in this study including: 1% YES (2% yeast extract, 1% sucrose), 15% YES (2% yeast extract, 15% sucrose), 1% YEP (2% yeast extract, 1% peptone), and 15% YEP (2% yeast extract, 15% peptone). The YES media is a toxin-conducive media and the YEP media is a toxin non-conducive media [36]. The concentrations of the media components were chosen based on optimized conditions determined by Davis *et al.* [34].

The media were supplemented with a gradient of hydrogen peroxide (H_2_O_2_; 3% stabilized solution) ranging from 0 to 50 mM. The media were then transferred to sterile 125 mL Erlenmeyer flasks with a total volume of 50 mL. The cultures were then inoculated with 100 μL of conidial suspension (~4.0 × 10^6^ conidia/mL) from their respective isolates and plugged with a sterile cotton ball. The cultures were then incubated under stationary conditions at 32 °C in the dark for 7 days.

Following incubation, the cultures were removed and the fungal mycelia were collected from each culture by filtration through non-sterilized Whatman no. 1 filter paper (GE Healthcare Life Sciences, Pittsburgh, PA, USA). The collected mycelia were then dried at 80 °C for 3–5 days and their dry biomass was recorded. In addition, a fraction of the culture filtrates were collected into amber glass vials and stored at 4 °C for use in aflatoxin measurement. Each assay was repeated at least three times.

### 3.3. Aflatoxin Measurement

The collected culture filtrates were then used to visualize the production of aflatoxin by the isolates in each medium and H_2_O_2_ combination using a modified thin layer chromatography (TLC) method based on that employed by Guo *et al.* [37]. Briefly, 500 μL of each culture filtrate was mixed with an equal volume of benzene in a 2 mL microfuge tube. The solution was then vortexed vigorously for 30 s. and then centrifuged at 10,000 × g at 25 °C for 2 min. The organic supernatant was then transferred to a 7 mL amber glass vial and was allowed to evaporate. The aflatoxin-containing residue was then dissolved in 100 μL methylene chloride and mixed by gentle inversion. For TLC analysis, 10 µL of each aflatoxin extract were spotted onto a silica-coated plate (10 × 20 cm; Z185329; Sigma Aldrich, St. Louis, MO, USA) along with an aflatoxin B_1_ standard (A6636; Sigma Aldrich, St. Louis, MO, USA). The plates were then developed in diethyl ether: methanol: water (96:3:1) and allowed to dry under the fume hood. The presence or absence of aflatoxin was then confirmed by viewing the plates under UV light (365 nm). The plates were then photographed using a Nikon Coolpix L110 digital camera (Nikon, Tokyo, Japan).

### 3.4. Hydrogen Peroxide Degradation Assay

In order to evaluate the potential for the supplemented H_2_O_2_ to be inactivated by the culture media carbon sources, the concentration of H_2_O_2_ within non-inoculated culture media was monitored over time. Briefly, each medium (1% YES, 15% YES, 1% YEP, and 15% YEP) and a water control were supplemented with H_2_O_2_ to a concentration of 30 mM. The supplemented media were then placed at 32 °C in the dark, the same conditions employed in the culture experiment. The levels of H_2_O_2_ were then measured every 24 h for 72 h using an Amplex Red hydrogen peroxide/peroxidase kit (Life Technologies, Carlsbad, CA, USA) according to the manufacturer’s instructions. A standard curve based on reaction H_2_O_2_ concentration was generated in order to calculate the H_2_O_2_ levels present in the culture media (Figure 2a). Fluorescence measurements performed during the assay were done using a Biotek Synergy HT plate reader (Biotek, Winooski, VT, USA). The assay was repeated twice for each of three biological replicates.

## 4. Conclusions

The contamination of agricultural crops with aflatoxins is a major concern for global food security, particularly in developing countries. Given that abiotic stresses such as drought stress have been shown to exacerbate the contamination of staple food crops such as maize, it is vital to understand the role of potentially causative chemical signals in this stress interaction. Since H_2_O_2_ is produced by plants in response to both pathogen infection and abiotic stress [33], a H_2_O_2_ gradient was used to simulate abiotic stress in toxigenic and atoxigenic isolates of *A. flavus* and *A. parasiticus* in order to examine the effects of hydrogen peroxide on fungal growth and aflatoxin production.

Toxigenic and atoxigenic isolates exhibited different levels of oxidative stress tolerance with toxigenic isolates generally surviving higher levels of oxidative stress than atoxigenic isolates. The production of aflatoxin in toxigenic isolates was also stimulated in response to increasing levels of oxidative stress. In addition, the elite atoxigenic biological control isolates of *A. flavus* were able to tolerate greater levels of H_2_O_2_-induced oxidative stress than other tested atoxigenic isolates. This indicates that this H_2_O_2_ gradient method may have utility in screening local atoxigenic isolates for use as biological control agents in developing countries due to the low cost and low degree of technical difficulty. We also found that peptone may react with H_2_O_2_ and reduce the effects of oxidative stress when used as a carbon source in culture media.

By better understanding the signals involved the responses of *A. flavus* and other *Aspergilli* to their environment; it may be possible to identify the key mechanisms involved in the host-pathogen interaction between *A. flavus* and staple food crops such as maize. This will allow for finding novel strategies for managing aflatoxin contamination, and for the enhancement of host resistance to aflatoxin contamination and abiotic stress through conventional breeding and biotechnology applications.
